# Emergence of topological and topological crystalline phases in TlBiS_2_ and TlSbS_2_

**DOI:** 10.1038/srep08379

**Published:** 2015-02-11

**Authors:** Qingyun Zhang, Yingchun Cheng, Udo Schwingenschlögl

**Affiliations:** 1 PSE Division, KAUST, Thuwal 23955-6900, Kingdom of Saudi Arabia

## Abstract

Using first-principles calculations, we investigate the band structure evolution and topological phase transitions in TlBiS_2_ and TlSbS_2_ under hydrostatic pressure as well as uniaxial and biaxial strain. The phase transitions are identified by parity analysis and by calculating the surface states. Zero, one, and four Dirac cones are found for the (111) surfaces of both TlBiS_2_ and TlSbS_2_ when the pressure grows, which confirms trivial-nontrivial-trivial phase transitions. The Dirac cones at the 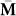 points are anisotropic with large out-of-plane component. TlBiS_2_ shows normal, topological, and topological crystalline insulator phases under hydrostatic pressure, thus being the first compound to exhibit a phase transition from a topological to a topological crystalline insulator.

Topological insulators, a new kind of quantum matter with an energy gap in the bulk and an odd number of gapless edge or surface states, have been studied intensively in the last decade^1^,^2^,^3^,^4^. Metallic surface states, which originate from spin-orbit coupling, are topologically protected by time-reversal symmetry, making the materials interesting for spintronic and quantum computation applications. Many three dimensional topological insulators have been theoretically predicted and experimentally confirmed^5^,^6^, including Tl-based III-V-VI_2_ ternary chalcogenides^7^,^8^,^9^,^10^,^11^,^12^,^13^,^14^,^15^. Among these compounds, TlBiS_2_ is found to be a trivial semiconductor in experiment, whereas different first principles calculations have predicted it to be either trivial^15^ or nontrivial^13^, implying that the topological property is very sensitive to the structural details. It has been reported that TlSbS_2_ can be tuned from a trivial insulator into a topological semimetal by uniaxial strain^13^. In general, strain engineering has been a fruitful path to new topological systems ever since HgTe had been predicted to undergo a phase transition under strain^2^. Various studies have dealt with strain induced topological phase transitions^16^,^17^,^18^,^19^,^20^,^21^,^22^,^23^,^24^,^25^.

Lately interest is shifting to the socalled topological crystalline insulators^26^,^27^, which are different from standard topological insulators in that the gapless surface states are protected by mirror symmetry rather than by time-reversal symmetry. The protection thus persists even when time-reversal symmetry is broken, for example by a magnetic field or magnetic dopant, which strongly broadens the field of potential applications. Topological crystalline insulators are rare, having been confirmed experimentally only for the SnTe class of compounds^28^,^29^,^30^,^31^,^32^ as well as for Bi_2_Te_3_^33^. To evaluate the physics of topological crystalline insulators, it is therefore essential to search for additional cases where this phase is realized. In the present work we study TlBiS_2_ and TlSbS_2_ by first principles density functional theory and demonstrate that hydrostatic pressure as well as uniaxial and biaxial strain induce topological phase transitions in both systems. For TlBiS_2_ a topological crystalline insulator phase emerges under hydrostatic pressure. The nature of the different phases is confirmed by calculating the surface states.

## Results

**Figure 1:**
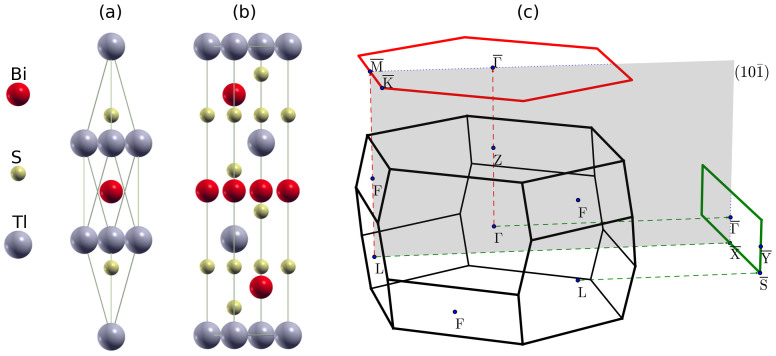
(a) Unit cell of TlBiS_2_, (b) corresponding hexagonal supercell, and (c) three dimensional Brillouin zone with projections onto the surface Brillouin zones.

TlBiS_2_ and TlBiS_2_ have rhombohedral structures with space group 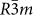, which is similar to Bi_2_Te_3_. There are four atoms per unit cell, with the Tl, Bi/Sb, and S atoms placed in layers normal to the three-fold axis in the sequence –Tl–S–Bi–S–, as shown in Fig. 1(a). Each Tl/Bi layer is sandwiched between two S layers, which results in a strong interlayer coupling so that the crystal structure is essentially three-dimensional. Tl, Bi, and S are located at the (0,0,0), (0.5,0.5,0.5), and (±*u*, ±*u*, ±*u*) sites, respectively. The structure has inversion symmetry where both Tl and Bi/Sb act as inversion centers. The corresponding hexagonal supercell is shown in Fig. 1(b) and the projections of the bulk Brillouin zone onto the surface Brillouin zones is demonstrated in Fig. 1(c). While hydrostatic pressure by definition is isotropic, the uniaxial strain is applied along the *z*-axis and the biaxial strain within the *xy*-plane. In no case the symmetry of the system is altered, which simplifies the analysis. The magnitude of the uniaxial strain is defined as 
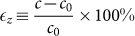
 and that of the biaxial strain as 
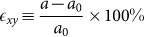
, where *a*_0_ and *c*_0_ are the in-plane and out-of-plane equilibrium lattice constants of the hexagonal supercell.

**Figure 2:**
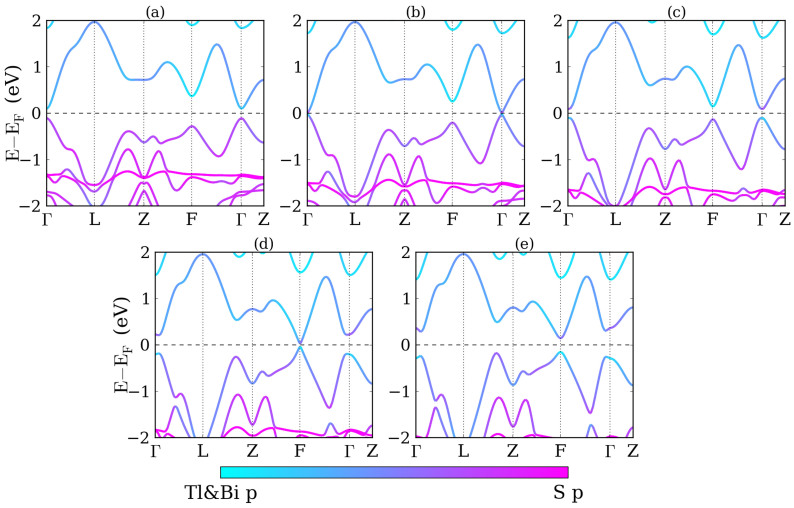
Band structures of TlBiS_2_ for (a) −2 GPa, (b) 0 GPa, (c) 2 GPa, (d) 5 GPa, and (e) 8 GPa hydrostatic pressure.

We first study the band structures and topological properties of TlBiS_2_ under strain. Structure relaxations are performed without spin-orbit coupling, yielding the lattice parameters *a*_0_ = 7.789 Å and angle *α* = 30.9°, which are close to the experimental values (*a*_0_ = 7.684 Å, *α* = 30.98°)^34^. The internal parameter characterizing the positions of the atoms is *u* = 0.237. The electronic band structure of TlBiS_2_ under hydrostatic pressure is addressed in Fig. 2. Without strain, see Fig. 2(b), the conduction band minimum and valence band maximum are both located at the Γ point with an energy gap of 7 meV. This value differs from previous calculations^13^,^15^,^35^, mainly due to different lattice constants, pseudopotentials, and exchange functionals. Examining the region near the Fermi energy, we find that the electronic properties are sensitive to the pressure. When the pressure increases from −2 GPa to 8 GPa the energy gap at the Γ point first closes and then reopens, with a critical pressure around 0 GPa. A similar behavior is found for the F point, with a critical pressure around 5 GPa. The system remains a direct band gap semiconductor up to 8 GPa, although the conduction band minimum and valence band maximum move from the Γ to the F point around 3 GPa. The band gaps for −2, 2, and 8 GPa pressure are 0.20, 0.19, and 0.28 eV, respectively. It is also found that at the conduction and valence band edges move away from the Γ point to the Γ-F line for pressure larger than 5 GPa. Finally, up to 15 GPa the band gaps at the Γ and F points are found to grow monotonously.

In Fig. 2 we project the wave functions to atomic orbitals, finding that the states around the Fermi level are mostly contributed by the Tl *p*, Bi *p*, and S *p* orbitals. In Fig. 2(a) the conduction bands are dominated by the Tl and Bi *p* orbitals, whereas the valence bands are dominated by the S *p* orbitals. No band inversion is found, which means that the system is in a trivial phase. In Fig. 2(b) the conduction and valence band edges touch each other and the orbital character switches at the Fermi level, i.e., a band inversion has occurred. In Fig. 2(d), for 5 GPa pressure, the conduction and valence bands touch at the F point and another band inversion occurs. Due to the even number of band inversions at the time-reversal invariant momenta the system is back to a trivial phase. The inversion symmetry of TlBiS_2_ allows us to perform a parity analysis. By checking the parities of the valence states at the eight time-reversal invariant momenta (1Γ, 3F, 1Z, and 3L), we find that the topological invariants at −2, 0, 2, 5 and 8 GPa pressure are (0;000), (1;000), (1;000), (0;000), and (0;000), respectively, consistent with the band inversion picture. We notice that the system is topologically nontrivial without strain, which contradicts the experimental result. The possible reason for this difference is the presence of strain in the sample due to the finite temperature, the substrate or other factors.

**Figure 3:**
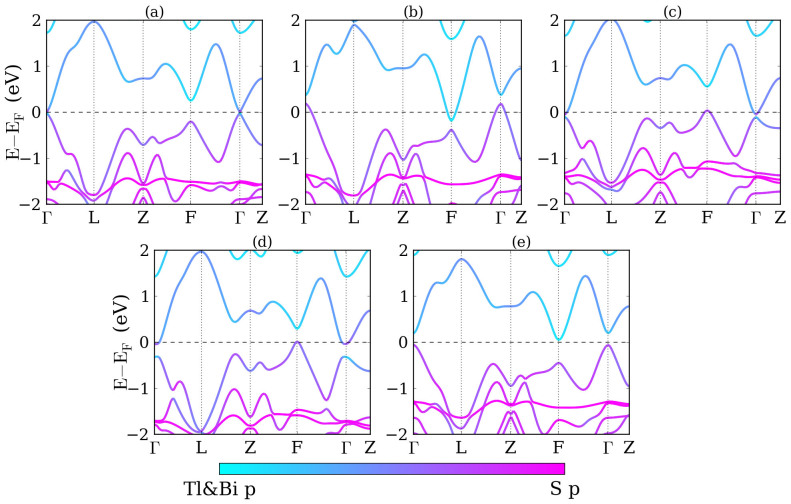
Band structures of TlBiS_2_ (a) without strain, with (b) −5%, (c) 5% biaxial strain as well as (d) −6% and (e) 6% uniaxial strain.

Band structures for biaxial and uniaxial strain are shown in Fig. 3(b)-(e), which are compared to the band structure without strain in Fig. 3(a). The variations under biaxial strain are very different from those under hydrostatic pressure. For biaxial compression, see Fig. 3(b), the system first changes from a direct gap semiconductor to an indirect gap semiconductor and then to a semimetal. No band inversion is found at the Γ and F points. On the other hand, Fig. 3(c) shows for biaxial tension that the energy gap at the Γ point first closes and then reopens. The conduction and valence band edges shift upwards at the F point and downwards at the Γ point, i.e., the system changes from a semiconductor to a semimetal. For uniaxial compression, see Fig. 3(d), a band inversion occurs at the Γ point. The system first turns into an indirect gap semiconductor and then into a semimetal. For uniaxial tension, see Fig. 3(e), no band inversion is found and the system remains an indirect gap semiconductor up to 6% strain. Using parity analysis, we find that the topological invariants are (0;000), (1;000), (1;000), and (0;000) under −5%, 5% biaxial strain and −6%, 6% uniaxial strain, respectively.

**Figure 4:**
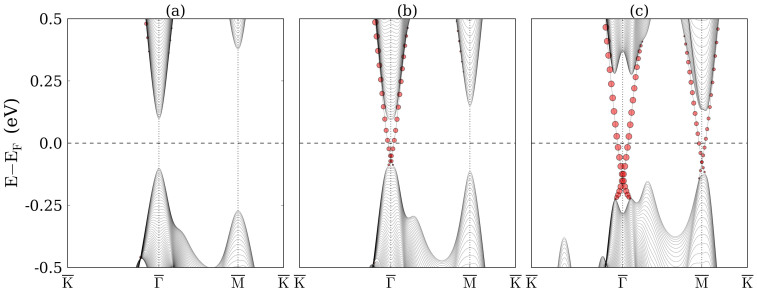
Band structures of the TlBiS_2_ slab with (111)-oriented surfaces under (a) −2 GPa, (b) 2 GPa, and (c) 5 GPa pressure. The size of the red circles represents the contribution of the first eight atomic layers.

To confirm the topological phase transitions, we investigate the evolution of the surface states of TlBiS_2_ under hydrostatic pressure. We first obtain a tight-binding Hamiltonian from maximally localized Wannier functions^36^ using the WANNIER90 code^37^. A hexagonal supercell with 8 × 8 × 8 k points is adopted in the non-self-consistent calculation. The Tl *s*, *p*, Bi *p*, and S *p* orbitals are used for the initial projection. Once the tight-binding Hamiltonian is established, a slab of 241 atomic layers with (111)-oriented surfaces is built with Tl on both the top and bottom surfaces. Calculated band structures near the Fermi level are shown in Fig. 4. Due to the inversion symmetry of the slab, the bands related to the top and bottom surfaces are degenerate. We project the states near the Fermi level to the first eight atomic layers on the top side of the slab. According to Fig. 4(a), for −2 GPa pressure there is no state in the bulk energy gap and the surface states marked by red circles are buried in bulk states. On the other hand, for 2 GPa pressure, see Fig. 4(b), a surface state emerges in the energy gap at the 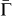 point and the Dirac point is located below the Fermi level, i.e., we have a nontrivial phase. For 8 GPa pressure, see Fig. 4(c), an additional surface state arises at the 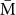 point. Since there are three 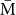 points in the surface Brillouin zone, we have a total of four Dirac cones. The even number again reflects a trivial phase. The contributions of the surface layers are highlighted by the size of the red circles. Comparing the surface states at the 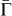 point in Fig. 4(b) and (c), we find that the proportion of the gapless states on the surface layers is determined by the pressure.

**Figure 5:**
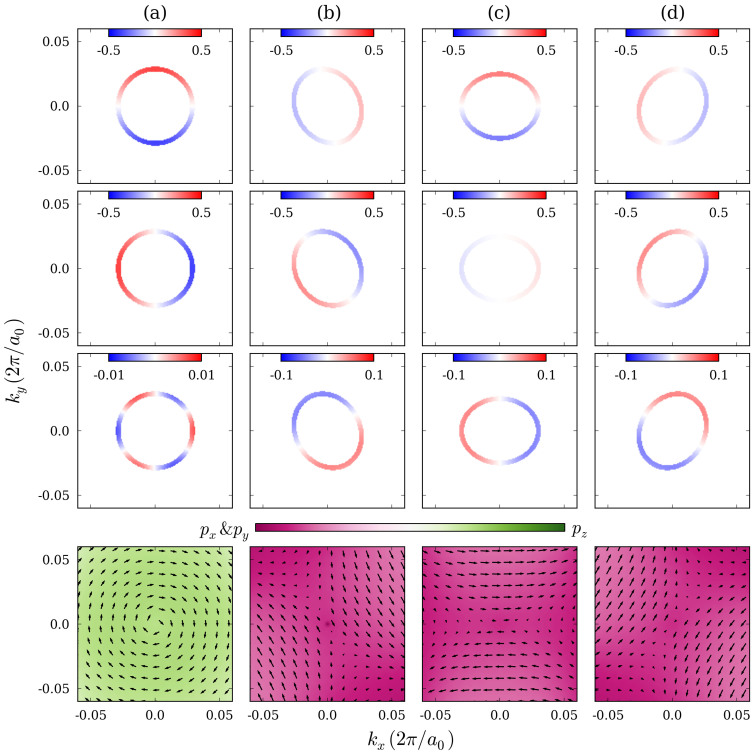
Spin polarization and orbital characteristics of the surface states of the TlBiS_2_ slab at the (a) 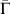, (b) 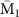, (c) 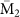, and (d) 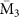 points under 8 GPa pressure. The first, second and third rows correspond to the spin *x*, *y* and *z* components at a constant energy. The fourth row shows the in-plane spin vectors and orbital characteristics above the Dirac point.

In Fig. 5 we show constant energy cuts through the Dirac cones for the 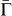 and 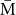 points at −0.04 eV and 0.02 eV, respectively. Clearly, the energy contour is anisotropic at the 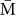 point with a distortion along the 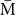-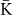 direction. Experimentally, above the Dirac point the spin direction is in-plane and precesses clockwise around the 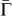 point, whereas below it precesses counterclockwise. In strained TlBiS_2_ we find the same property for the surface states. There is also a small *z* component with three fold symmetry, which results from the in-plane potential gradients^38^. These findings agree with previous calculations in Ref. 14. It is a distinct feature of the surface states at the 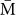 point that the spin amplitudes are different for the *x* and *y* components. The spin *z* component has no three fold symmetry and the amplitude is larger than at the 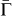 point. This larger deviation of the spin vector from the *xy*-plane results from the higher in-plane anisotropy at the 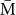 point. By fitting the data, we find that the in-plane spin angle *θ_s_* obeys the following relations with respect to the azimuthal angle *θ* of the momentum:
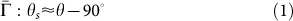

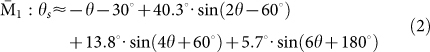

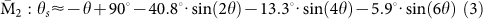

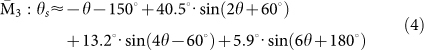
The direction of the spin and momentum are related to each other at the 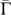 point, whereas at the 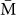 point the spin behaves inversely to the momentum and has a large out-of-phase component, which is due to the anisotropy of the Dirac cone. This relation between spin and momentum can be explained by the spin-orbital texture of the surface states which is also found in Bi_2_Se_3_ and Bi_2_Te_3_^39^,^40^. At the 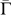 and 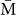 points the surface states are dominated by the out-of-plane *p_z_* and in-plane *p_x_*, *p_y_* orbitals, respectively.

**Figure 6:**
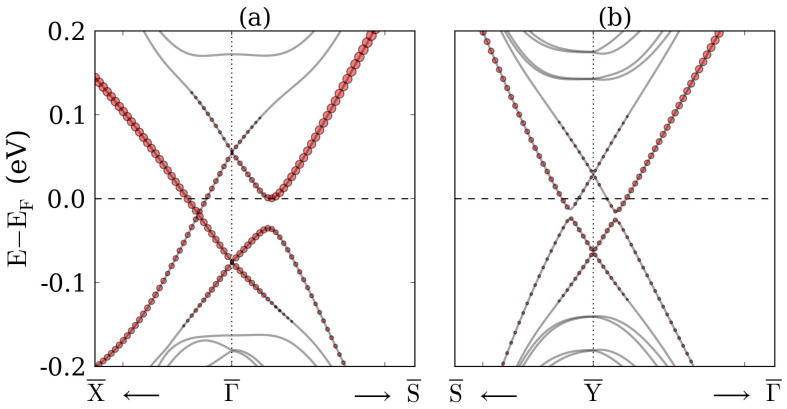
Band structure of the TlBiS_2_ slab with 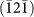-oriented surfaces in the vicinity of the (a) 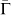 and (b) 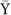 points. The slab consists of 90 atomic layers. The size of the red circles represents the contribution of the first three atomic layers.

Topological crystalline insulators are characterized by a non-zero mirror Chern number. Therefore, mirror symmetry protected Dirac cones arise in the surface electronic structure. Similarity between the surface states of TlBiS_2_ and SnTe, compare Refs. 28–32, 41, 42, implies that TlBiS_2_ becomes a topological crystalline insulator at 8 GPa pressure. To confirm this conjecture, we study the bands of the 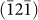 surface of TlBiS_2_, see Fig. 6, which is symmetrical about the 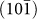 mirror plane. Figure 1(c) demonstrates that the 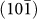 mirror plane crosses the surface Brillouin zone along the line 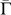-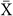 and that the Γ point as well as one of the F points of the bulk Brillouin zone are projected onto the 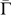 point. Since the band structure in Fig. 2(e) shows band inversion at both the Γ and F points, we reproduce the general situation of Ref. 41 and expect the projected Dirac cones to hybridize at their intersection. This expectation is confirmed by Fig. 6(a), which shows that the hybridization opens an energy gap at the Fermi level, except for the mirror-symmetric line 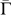-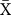. Along this line a descendent Dirac cone is created due to protection by mirror symmetry (rather than by time-reversal symmetry). At the 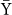 point, see Fig. 6(b), the hybridization leads to an energy gap, because the two F points that are projected onto this point do not lie on the 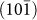 mirror plane^42^. To the best of our knowledge, TlBiS_2_ is the first reported compound which can be tuned from a topological insulator into a topological crystalline insulator by applying hydrostatic pressure.

**Figure 7:**
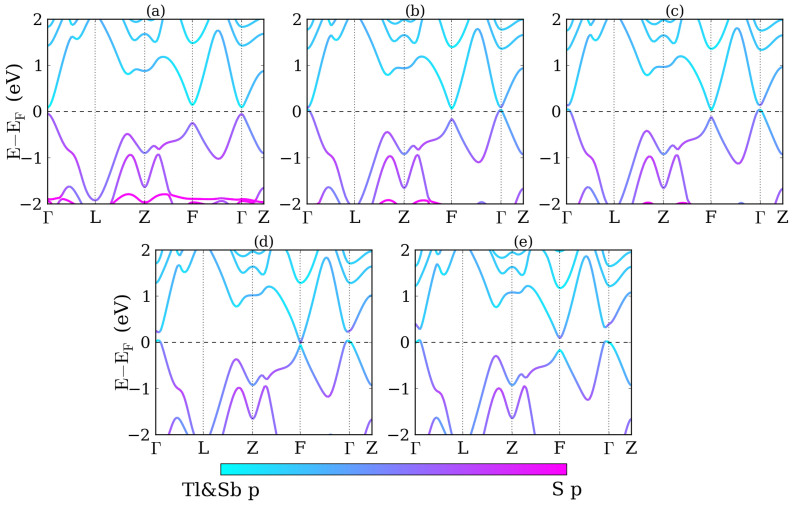
Band structures of TlSbS_2_ for (a) 0 GPa, (b) 2 GPa, (c) 3 GPa, (d) 5 GPa, and (e) 8 GPa hydrostatic pressures.

For TlSbS_2_ without strain we obtain the lattice parameters *a*_0_ = 7.758 Å and *α* = 30.3° as well as the internal parameter *u* = 0.236. Without strain, see Fig. 7(a), we obtain a direct gap semiconductor with an energy gap of 0.128 eV at the Γ point. When the pressure increases from 0 GPa to 8 GPa the energy gaps at the Γ and F points first close and then reopen, similar to TlBiS_2_. The critical pressures for closure are around 2 and 5 GPa for the Γ and F points, respectively. Distinctly different to TlBiS_2_, under pressure the valence band edge at the Γ point shifts upwards and the conduction band edge at the F point downwards, which makes the system change from a semiconductor to a semimetal. Similar to TlBiS_2_, the states around the Fermi level are mostly due to the Tl *p*, Sb *p*, and S *p* orbitals. In Fig. 7(a) we find no band inversions at the time-reversal invariant momenta and the conduction bands are dominated by the Tl and Sb *p* orbitals, while the valence bands are dominated by the S *p* orbitals. In Fig. 7(b) a band inversion occurs at the Γ point, i.e., the system changes from a trivial phase into a nontrivial phase. For 5 GPa pressure, see Fig. 7(d), the conduction and valence band edges touch at the F point and another band inversion occurs, transforming the system from a topological semimetal into a trivial semimetal. By parity analysis, we find that the topological invariants without pressure and under 2, 3, 5, and 8 GPa pressure are (0;000), (1;000), (1;000), (0;000), and (0;000), respectively.

**Figure 8:**
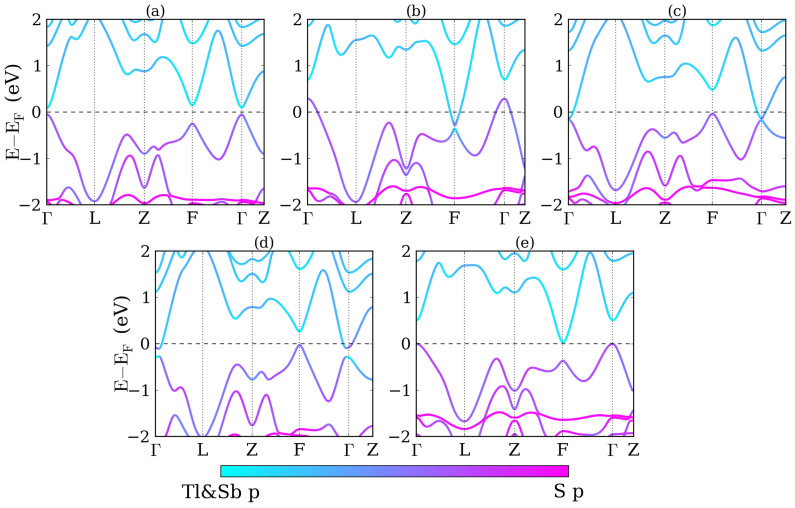
Band structures of TlSbS_2_ (a) without strain, with (b) −5%, (c) 5% biaxial strains as well as (d) −6%, and (e) 6% uniaxial strains.

Band structures under biaxial and uniaxial strain are illustrated in Fig. 8(b)–(e). For comparison, the band structure without strain is shown in Fig. 8(a). For biaxial compression, see Fig. 8(b), the system transforms from a direct gap semiconductor into a semimetal with the valence band maximum and conduction band minimum located at the Γ and F point, respectively. A band inversion occurs at the F point and the energy gap at the Γ point grows. According to Fig. 8(c), under biaxial tension the variation of the band structure near the Fermi level is reversed as compared to compression. We observe that at the F point the conduction and valence band edges shift upwards and the energy gap grows, whereas at the Γ point the band edges shift downwards and touch at 5% tension. For uniaxial compression, see Fig. 8(d), the system changes from a direct gap semiconductor into a semimetal with a band inversion at the Γ point, whereas for uniaxial tension in Fig. 8(e) it first changes from a direct gap semiconductor into an indirect gap semiconductor and then into a semimetal. The band gaps at the Γ and F points both grow and no band inversion appears. By parity analysis, we obtain the topological invariants (1;000), (1;000), (1;000), and (0;000) under −5%, 5% biaxial strain, and −6%, 6% uniaxial strain, respectively.

**Figure 9:**
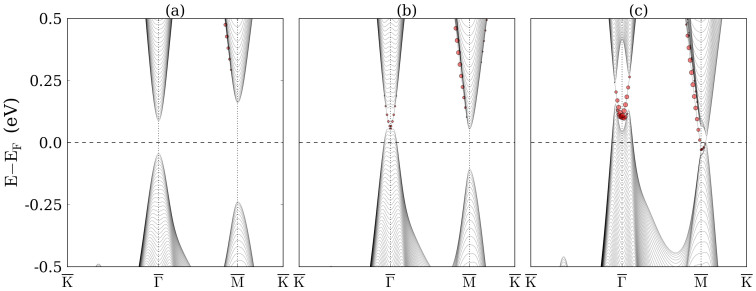
Band structures of the TlSbS_2_ slab under (a) 0 GPa, (b) 3 GPa, and (c) 5 GPa pressure. The size of the red circles represents the contribution of the surface layer.

The surface states are studied analogously to TlBiS_2_. Without strain, see Fig. 9(a), there is no surface state in the bulk energy gap, i.e., the system is a trivial semiconductor, whereas under 3 GPa pressure, see Fig. 9(b), a surface state emerges in the energy gap at the 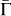 point above the Fermi level. The system is in a nontrivial phase and the Dirac cone is buried in bulk states. Under 8 GPa pressure, see Fig. 9(c), an additional surface state arises at the 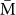 point, located at the Fermi level, but still buried in bulk states. TlSbS_2_ under 8 GPa pressure is a topological crystalline semimetal. Due to the semimetallic nature, the surface states here are less interesting than for TlBiS_2_, although the spin-orbital textures of the Dirac cones are similar.

## Discussion

We have studied the topological properties of TlBiS_2_ and TlSbS_2_ under strain. Two topological phase transitions occur in both systems under hydrostatic pressure. TlBiS_2_ remains a direct gap semiconductor up to 8 GPa, while TlSbS_2_ turns from a semiconductor into a semimetal. Biaxial and uniaxial strain also drive topological phase transitions. Band structures of slabs with (111)-oriented surfaces show that a surface state emerges at the 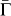 point when the system is in the topological regime. For pressure larger than 5 GPa another surface state appears at the 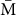 point, transforming the system from a nontrivial into a trivial phase. By further investigating the states on the 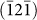 surface, which are symmetric about the 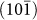 mirror plane, we find that TlBiS_2_ under 8 GPa pressure undergoes a phase transition from a topological to a topological crystalline insulator. Similar transitions can also be expected for other Tl-based III-V-VI_2_ ternary chalcogenides, for example TlBiTe_2_. The surface states induced by strain should be investigated by spin- and angle-resolved photoemission spectroscopy to confirm our predictions.

## Methods

Our calculations are based on the Quantum ESPRESSO code^43^, using the generalized gradient approximation for the exchange-correlation functional and norm-conserving pseudopotentials. A kinetic energy cutoff of 680 eV and a 10 × 10 × 10 k-point mesh are employed.

## Author Contributions

Q.Y.Z. performed the calculations. Y.C.C. and U.S. contributed to the analysis. All authors wrote the manuscript.

## References

[b1] Kane C. L. & Mele E. J. Z_2_ topological order and the quantum spin Hall effect. Phys. Rev. Lett. 95, 146802 (2005). 16241681 10.1103/PhysRevLett.95.146802

[b2] Fu L. & Kane C. L. Topological insulators with inversion symmetry. Phys. Rev. B 76, 045302 (2007).

[b3] Hasan M. Z. & Kane C. L. Topological insulators. Rev. Mod. Phys. 82, 3045–3067 (2010).

[b4] Qi X.-L. & Zhang S.-C. Topological insulators and superconductors. Rev. Mod. Phys. 83, 1057–1110 (2011).

[b5] Yan B. H. & Zhang S.-C. Topological materials. Rep. Prog. Phys. 75, 096501 (2012). 22907264 10.1088/0034-4885/75/9/096501

[b6] Feng W. X. & Yao Y. G. Three-dimensional topological insulators: A review on host materials. Sci. China Phys. Mech. Astron. 55, 2199 (2012).

[b7] Kuroda K. *et al.* Experimental realization of a three-dimensional topological insulator phase in ternary chalcogenide TlBiSe_2_. Phys. Rev. Lett. 105, 146801 (2010). 21230855 10.1103/PhysRevLett.105.146801

[b8] Chen Y. L. *et al.* Single Dirac cone topological surface state and unusual thermoelectric property of compounds from a new topological insulator family. Phys. Rev. Lett. 105, 266401 (2010). 21231687 10.1103/PhysRevLett.105.266401

[b9] Sato T. *et al.* Unexpected mass acquisition of Dirac fermions at the quantum phase transition of a topological insulator. Nat. Phys. 7, 840–844 (2011).

[b10] Xu S.-Y. *et al.* Topological phase transition and texture inversion in a tunable topological insulator. Science 332, 560–564 (2011). 21454752 10.1126/science.1201607

[b11] Souma S. *et al.* Spin polarization of gapped Dirac surface states near the topological phase transition in TlBi(S_1−*x*_Se*_x_*)_2_. Phys. Rev. Lett. 109, 186804 (2012). 23215312 10.1103/PhysRevLett.109.186804

[b12] Lin H. *et al.* Single-Dirac-cone topological surface states in the TlBiSe_2_ class of topological semiconductors. Phys. Rev. Lett. 105, 036404 (2010). 20867784 10.1103/PhysRevLett.105.036404

[b13] Yan B. H. *et al.* Theoretical prediction of topological insulators in thallium-based III-V-VI_2_ ternary chalcogenides. Europhys. Lett. 90, 37002 (2010).

[b14] Eremeev S. V. *et al.* Ab initio electronic structure of thallium-based topological insulators. Phys. Rev. B 83, 205129 (2011).

[b15] Singh B. *et al.* Topological electronic structure and Weyl semimetal in the TlBiSe_2_ class of semiconductors. Phys. Rev. B 86, 115208 (2012).

[b16] Feng W. *et al.* Strain tuning of topological band order in cubic semiconductors. Phys. Rev. B 85, 195114 (2012).

[b17] Winterfeld L. *et al.* Strain-induced topological insulator phase transition in HgSe. Phys. Rev. B 87, 075143 (2013).

[b18] Agapito L. A., Kioussis N., Goddard III W. A. & Ong N. P. Novel family of chiral-based topological insulators: elemental tellurium under strain. Phys. Rev. Lett. 110, 176401 (2013). 23679748 10.1103/PhysRevLett.110.176401

[b19] Zhang Q., Cheng Y. & Schwingenschlögl U. Series of topological phase transitions in TiTe_2_ under strain. Phys. Rev. B 88, 155317 (2013).

[b20] Bahramy M., Yang B.-J., Arita R. & Nagaosa N. Emergence of non-centrosymmetric topological insulating phase in BiTeI under pressure. Nat. Commun. 3, 679 (2012). 22334082 10.1038/ncomms1679

[b21] Zhu Z. Y., Cheng Y. C. & Schwingenschlögl U. Topological phase transition in layered GaS and GaSe. Phys. Rev. Lett. 108, 266805 (2012). 23005005 10.1103/PhysRevLett.108.266805

[b22] Sun Y. *et al.* Strain-driven onset of nontrivial topological insulating states in Zintl Sr_2_X compounds (X = Pb, Sn). Phys. Rev. B 84, 165127 (2011).

[b23] Sun Y., Chen X-Q., Yunoki S., Li D. & Li Y. New family of three-dimensional topological insulators with antiperovskite structure. Phys. Rev. Lett. 105, 216406 (2010). 21231331 10.1103/PhysRevLett.105.216406

[b24] Young S. M. *et al.* Theoretical investigation of the evolution of the topological phase of Bi_2_Se_3_ under mechanical strain. Phys. Rev. B 84, 085106 (2011).

[b25] Sa B., Zhou J., Song Z., Sun Z. & Ahuja R. Pressure-induced topological insulating behavior in the ternary chalcogenide Ge_2_Sb_2_Te_5_. Phys. Rev. B 84, 085130 (2011).

[b26] Teo J. C. Y., Fu L. & Kane C. L. Surface states and topological invariants in three-dimensional topological insulators: Application to Bi_1−*x*_Sb*_x_*. Phys. Rev. B 78, 045426 (2008).

[b27] Fu L. Topological crystalline insulators. Phys. Rev. Lett. 106, 106802 (2011). 21469822 10.1103/PhysRevLett.106.106802

[b28] Hsieh T. H. *et al.* Topological crystalline insulators in the SnTe material class. Nat. Commun. 3, 982 (2012). 22864575 10.1038/ncomms1969

[b29] Tanaka Y. *et al.* Experimental realization of a topological crystalline insulator in SnTe. Nat. Phys. 8, 800 (2012).

[b30] Dziawa P. *et al.* Topological crystalline insulator states in Pb_1−*x*_Sn*_x_*Se. Nat. Mater. 11, 1023 (2012). 23023551 10.1038/nmat3449

[b31] Xu S.-Y. *et al.* Observation of a topological crystalline insulator phase and topological phase transition in Pb_1−*x*_Sn*_x_*Te. Nat. Commun. 3, 1192 (2012). 23149737 10.1038/ncomms2191

[b32] Wojek B. M. *et al.* Spin-polarized (001) surface states of the topological crystalline insulator Pb_0.73_Sn_0.27_Se. Phys. Rev. B 87, 115106 (2013).

[b33] Rauch T., Flieger M., Henk J., Mertig I. & Ernst A. Dual topological character of chalcogenides: theory for Bi_2_Te_3_. Phys. Rev. Lett. 112, 016802 (2014). 24483917 10.1103/PhysRevLett.112.016802

[b34] Özer M., Paraskevopoulos K. M., Anagnostopoulos A. N., Kokou S. & Polychroniadis E. K. Large single-crystal growth and characterization of the narrow-gap semiconductor TlBiS_2_. Semicond. Sci. Technol. 11, 1405 (1996).

[b35] Hoang K. & Mahanti S. D. Atomic and electronic structures of thallium-based III-V-VI_2_ ternary chalcogenides: Ab initio calculations. Phys. Rev. B 77, 205107 (2008).

[b36] Marzari N. & Vanderbilt D. Maximally localized generalized Wannier functions for composite energy bands. Phys. Rev. B 56, 12847 (1997).

[b37] Mostofi A. A. *et al.* wannier90: A tool for obtaining maximally-localised Wannier functions. Comput. Phys. Commun. 178, 685 (2008).

[b38] Premper J., Trautmann M., Henk J. & Bruno P. Spin-orbit splitting in an anisotropic two-dimensional electron gas. Phys. Rev. B 76, 073310 (2007).

[b39] Cao Y. *et al.* Mapping the orbital wavefunction of the surface states in three-dimensional topological insulators. Nat. Phys. 9, 499–504 (2013).

[b40] Zhang H.-J., Liu C.-X. & Zhang S.-C. Spin-orbital texture in topological insulators. Phys. Rev. Lett. 111, 066801 (2013). 23971598 10.1103/PhysRevLett.111.066801

[b41] Liu J., Duan W. & Fu L. Two types of surface states in topological crystalline insulators. Phys. Rev. B 88, 241303(R) (2013).

[b42] Safaei S., Kacman P. & Buczko R. Topological crystalline insulator (Pb,Sn)Te: Surface states and their spin polarization. Phys. Rev. B 88, 045305 (2013).

[b43] Giannozzi P. *et al.* QUANTUM ESPRESSO: a modular and open-source software project for quantum simulations of materials. J. Phys.: Condens. Matter 21, 395502 (2009). 21832390 10.1088/0953-8984/21/39/395502

